# Effects of COVID-19-related life changes on mental health in Syrian refugees in Turkey

**DOI:** 10.1192/bjo.2021.1009

**Published:** 2021-10-01

**Authors:** Luca Bernardi, Ian H. Gotlib, Özge Zihnioğlu

**Affiliations:** Department of Politics, University of Liverpool, UK; Department of Psychology, Stanford University, USA; Department of Politics, University of Liverpool, UK

**Keywords:** Depressive disorders, anxiety disorders, post-traumatic stress disorder, stigma and discrimination, low- and middle-income countries

## Abstract

**Background:**

Mental disorders are currently the greatest global health burden. The coronavirus diseases 2019 (COVID-19) pandemic is having an adverse impact on people's mental health, particularly in vulnerable populations, such as refugees.

**Aims:**

The present study was designed to examine the association between COVID-19 and changes in mental health in Syrian refugees in Turkey.

**Method:**

We conducted a two-wave panel survey of a representative sample of 302 of the estimated 500 000 Syrian refugees (ages 18 and older) living under humanitarian support in Istanbul (first wave between 9 and 15 July 2020 and the follow-up between 11 and 14 September 2020). We administered seven items from the CoRonavIruS Health Impact Survey in addition to one-context specific item about life changes because of COVID-19, and measures of depression (10-item Center for Epidemiologic Study Depression Scale, CESD-10), anxiety (6-item State-Trait Anxiety Inventory, STAI-6) and perceived stress (Perceived Stress Scale, PSS-4).

**Results:**

A factor analysis yielded three COVID-19 factors, labelled ‘social relationships’, ‘stress’ and ‘hope.’ We conducted a series of cross-lag panel analyses to test associations between the COVID-19 factors and mental health. We found associations between all COVID-19 factors and CESD-10, between COVID-19 ‘stress’ and STAI-6, and between COVID-19 ‘stress’ and COVID-19 ‘hope’ and PSS-4.

**Conclusions:**

Our measures of life changes because of the COVID-19 pandemic are associated with changes in the mental health of Syrian refugees living in Istanbul. It is therefore important that they are provided with services to reduce what may be particularly debilitating consequences of COVID-19.

## Background

Mental disorders are the greatest global health burden: each year, one in four individuals will experience at least one diagnosable mental health problem.^1^ Depression and anxiety are particularly prevalent; in fact, according to the World Health Organization (WHO), depression is the leading cause of disability worldwide and is projected to become the world's most burdensome disease in the coming decades.^2^

The measures introduced by governments around the world to limit the spread and contagion of coronavirus disease 2019 (COVID-19) are being found to have critical implications for mental health,^3–6^ prompting calls for action concerning research examining the impact of COVID-19 on mental health.^7,8^ Not surprisingly, the COVID-19 pandemic may be particularly challenging for more vulnerable populations such as refugees, whose traumatic experiences have already been found to significantly affect their mental health.^9^ Indeed, the prevalence of psychiatric problems in refugees is particularly high. Although there is considerable variation across refugee populations and assessment measures, a meta-analysis of articles published between 1998 and 2018 documented high lifetime prevalence rates of post-traumatic stress disorder (PTSD), depression and anxiety.^10^

Our study focuses on Syrian refugees living under temporary protection in Turkey. Since the outbreak of the Syrian civil war in 2011, Turkey has become home to one of the largest refugee populations in the world; in fact, over 3.6 million Syrians live in Turkey under temporary protection.^11^

Temporary protection status grants Syrian refugees access to public services such as healthcare, education and the labour market. Experts estimate that between 500 000 to 1 000 000 Syrians work in Turkey.^12^ Registered Syrians in Turkey have been able to apply for a work permit since 2016 – according to the Minister of Interior, only 65 000 Syrians have a work permit;^13^ however, work permits are not only costly and time consuming to obtain, but they must also be renewed annually. Further, even with a valid work permit Syrians are not eligible to receive cash support through the Emergency Social Safety Net.^14^ Lack of language skills and difficulties in transferring university degrees further limit formal employment opportunities for refugees.

These restrictions push refugees to take informal jobs in unskilled services and industry.^14^ A study by the International Labour Organization estimates more than 97% of Syrians work informally in Turkey.^15^ These jobs have become especially precarious with on-and-off measures restricting businesses. Whereas Syrian refugees’ problems regarding working conditions, accommodation and discrimination decreased between 2017 and 2019, this is likely to have changed since mid-2020 because of lockdown measures and other restrictions making it more difficult to work during the pandemic.^16^ Finally, tensions between Turkish and Syrian communities and significant uneasiness in particular of the Turkish community towards the Syrians has become more visible over the past few years. Although recent research reports a high level of social acceptance of the refugees, it is not clear how robust this is.^16^

Hence, although researchers have provided prevalence estimates of mental health problems such as PTSD, depression and anxiety in Syrian refugees in various countries – including Turkey, Sweden, Germany and the USA and have investigated the effect of pre-migration, migration and post-migration factors,^17–22^ we know much less about how the COVID-19 pandemic is contributing to a worsening of mental health in refugees.^23–27^ The present study is the first, to our knowledge, to analyse the effect of the COVID-19 pandemic on the mental health of Syrian refugees in Istanbul.

## Aims

Our goal is to use our two-wave Syrian Refugee Mental Health Panel Study to examine the association between COVID-19 factors and mental health. Given recent findings of a bidirectional relationship between COVID-19 and psychiatric disorders,^4^ it is important to study not only whether the pandemic is associated with mental health deterioration but also whether the latter may act as a susceptibility or vulnerability factor for maintenance of pandemic-related stressors. That is, the pandemic may have negatively affected Syrian refugees’ mental health, which may in turn have sustained or increased their worries about their social context, financial situation, and employment status and about antipandemic restrictions.

We set out to conduct a series of cross-lag models assessing the relationship among symptoms of depression, anxiety and perceived stress to test whether life changes because of COVID-19 are associated with changes in the mental health of Syrian refugees living in Istanbul. Given the consistent findings of gender differences in depression,^28^ which was also supported in some research with Syrian refugees,^17,19,21^ we also set out to test for an interaction between COVID-19 factors and gender. Finally, using our two-wave panel data we set out to examine the bidirectional nature of the association between COVID-19 factors and symptoms of mental illness.

## Method

### Study design, setting and participant recruitment

We conducted a two-wave panel survey (with computer-assisted telephone interview methodology) of a representative sample of 302 of the estimated 500 000 Syrian refugees in Istanbul.^11^ We worked with the polling firm Frekans Research, which has extensive experience conducting research with Syrian refugees. Frekans Research has a database that includes the contact information for a representative sample of 1200 Syrian refugees in Istanbul. This sample was selected randomly based on government statistics about the residential area Syrian refugees live in. The sample for the current study was randomly selected from that representative sample of 1200 refugees in Istanbul who completed face-to-face interviews and gave their consent to participate in future studies.

The participants included adult refugees (aged 18 year or older) living under temporary protection in Turkey, but not in camps. Frekans research has validated our questionnaire for Syrian refugees. Professional translators at Frekans Research translated our questionnaire in parallel into both Turkish and Arabic. The questionnaire was back translated into English as well. We conducted the first wave of data collection between 9 and 15 July 2020 (*n* = 302), and the follow-up 2 months later between 11 and 14 September 2020 (*n* = 210); the response rate for the follow-up was 70%. Of our 302 Syrian refugees, 50% are females, mean age is 32 (range 18–69), 34% have primary school, 39% middle school, 20% high school and 7% master/doctorate education. The mean total household income per month was 2536 Turkish Liras, 17% were unemployed, 28% had reported to have experienced discrimination in the past 12 months, 47% have reported being very or extremely concerned about the stability of their residential status in Turkey and the average number of years living in Istanbul was 5.

The authors assert that all procedures contributing to this work comply with the ethical standards of the relevant national and institutional committees on human experimentation and with the Helsinki Declaration of 1975, as revised in 2008. More specifically, Frekans Research is a member of European Society for Opinion and Marketing Research and the Turkish Research Association and was awarded the Trustworthy Research Certificate, and so they abide with the code of conduct of these committees. All procedures involving human participants were approved by the ethics committee of the School of Histories, Languages and Cultures of the University of Liverpool, approval number 7866.

Before starting the survey, the interviewers asked participants to give their informed consent by reading the consent wording to each participant, briefly explaining the nature of the study, the approximate duration of the survey, and informing the individuals that participation in the survey was voluntary and that their confidentiality was protected. The data were fully anonymised after the fieldwork and individual ID numbers were created that were used to link respondents’ answers in the follow-up wave. All analyses were conducted with Stata 14.

### Measures

Our questionnaire integrates a selection of questions about life changes because of COVID-19 from the CoRonavIruS Health Impact Survey (CRISIS) developed by the National Institute of Mental Health (http://www.crisissurvey.org). More specifically, we used seven items from the CRISIS questionnaire section on ‘Life changes due to Coronavirus/COVID-19 crisis in the last two weeks’ integrated by one context-specific item about the quality of relationships with the Turkish community (see Appendix).

The CRISIS questionnaire asks about respondents’ feelings concerning a number of COVID-19-related changes in their daily life, including stress about restrictions of leaving home, change in the quality of social relationships, concerns about the financial situation and feelings of hope towards the end of the pandemic. The rationale of the item selection meets three criteria: budget constraints, survey timing and context. As our surveys were conducted in the middle of the pandemic and not at the beginning and as some of those items were US-specific, our team has selected a subset of items capturing COVID-19 stressors that could be used in later phases of the pandemic and outside the USA. The Appendix reports the full list of COVID-19 questions used in this research along with their response options and scoring range. In the analyses, responses for questions on the quality of the relationships and hopefulness for the end of the pandemic were reversed so that higher values denote more negative or problematic responses.

We used three validated measures of symptoms of mental disorders to assess Syrian refugees’ mental health. All measures were coded so that higher values reflect higher symptoms. In Supplementary Appendix A available at https://doi.org/10.1192/bjo.2021.1009 we present the full question wordings and response options of these questionnaires. For symptoms of depression we used the 10-item Center for Epidemiologic Study Depression Scale (CESD-10).^29^ The depression scale is a shorter 10-item form of the CES-D. Respondents were asked to indicate how often or how consistently they have felt or behaved this way during the past 2 weeks: I was bothered by things that usually don't bother me; I had trouble keeping my mind on what I was doing; depressed; I felt that everything I did was an effort; I felt hopeful about the future; I felt fearful; My sleep was restless; I was happy; I felt lonely; I could not get ‘going’. Response options were: 0, rarely or none of the time (less than 1 day); 1, some or a little of the time (1–2 days); 2, occasionally or a moderate amount of time (3–4 days); 3, most or all of the time (5–7 days).

For symptoms of anxiety, we used the 6-item State-Trait Anxiety Inventory (STAI-6).^30^ Respondents were asked to state how they have been feeling in the past 2 weeks on the following items: I have been feeling calm; I have been feeling tense; I have been feeling relaxed; I have been feeling upset; I have been feeling content; I have been feeling worried. Response options were: 0, never; 1, sometimes; 2, often; 3, almost always.

For symptoms of stress, we used the 4-item Perceived Stress Scale (PSS-4).^31^ Respondents were asked about their feelings and thoughts in the past 2 weeks: How often have you felt that you were unable to control the important things in your life? How often have you felt confident about your ability to handle your personal problems? How often have you felt that things were going your way? How often have you felt difficulties were piling up so high that you could not overcome them? Response options were: 0, never; 1, almost never; 2, sometimes; 3, fairly often; 4, very often.

In the analyses we included a number of pre-migration and post-migration factors for which we asked for information in our surveys: gender, age, education, employment status, total monthly household income, number of years living in Istanbul, whether experienced discrimination, perception of Turkish society's negative attitude towards Syrian refugees, and concern about stability of residential status in Turkey. Descriptive statistics for all variables are presented in Supplementary Appendix B. To maximise comparability of questions across Syrian refugee surveys, we asked many of the above questions in the same way as they were asked in the Syrian Refugee Panel Study,^32^ which examines the integration of Syrian refugees into host European societies.

### Statistical analysis

We first conducted a principal components factor analysis with varimax orthogonal rotation to derive COVID-19 factor scores. Rotation is a common procedure in that, by forcing factors to be uncorrelated, it reduces ambiguity about which item belongs to which factor. Next, we took advantage of the longitudinal nature of our data and conducted a series of cross-lagged panel analyses based on linear regression models with maximum likelihood random-effects estimator to explore the causal relationship between COVID-19 factors and mental health.

Thus, in the models that estimate the effect of COVID-19 factors on measures of mental health at time (*t* – 1), we include levels of COVID-19 factors and mental health at time (*t*). An example of such models is presented in Equation (1), where CESD-10 (*t*) is the dependent variable. We estimate the same model for STAI-6 and PSS-4:1
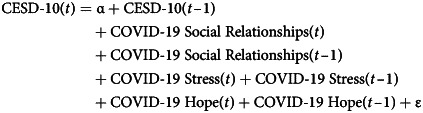


Thus, Equation (1) models depressive symptoms at time (*t*) as a function of depressive symptoms at time (*t* – 1), and COVID-19 factors (i.e. social relationships, stress and hope) at both time (*t*) and (*t* – 1), where α is the intercept and ε the error term.

Similarly, in the models that estimate the effects of mental health on COVID-19 factors at time (*t* – 1), we include COVID-19 factors along with mental health at time (*t*). Thus, in these analyses COVID-19 factors are the dependent variables and we estimate these models separately for each of our three COVID-19 factors. In these analyses, we also control for the two other two COVID-19 factors at both time (*t*) and (*t* – 1). An example of such models is presented in Equation (2), where COVID-19 social relationships (*t*) is the dependent variable:2
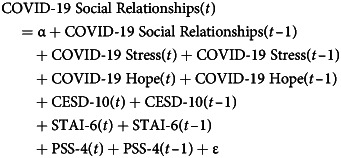


Thus, Equation (2) models COVID-19 social relationships at time (*t*) as a function of COVID-19 social relationships at time (*t* – 1), the other two COVID-19 factors at both time (*t*) and (*t* – 1), and our three mental health measures at both time (*t*) and (*t* – 1). For simplicity, the basic models reported in Equations (1) and (2) omit all sociodemographic variables included in the analyses.

The longitudinal nature of our data allowed us to examine the bidirectional nature of the association between COVID-19 factors and symptoms of mental illness. That is, COVID-19 factors could adversely affect mental health, and poorer mental health could sustain or increase pandemic-related worries and stresses. Akaike's Information Criterion (AIC) is a goodness of fit measure that can be used to examine this question^33,34^ by comparing alternative models. The model with the smallest AIC value fits the data better and, therefore, represents the best balance of goodness of fit and complexity. Finally, in supplementary analyses we test for an interaction between gender and COVID-19 factors to see whether the pandemic has affected Syrian refugees’ mental health differently for males and females.

## Results

Before moving to the results from our regression analyses, we report the findings from our rotated factor analysis used to derive our COVID-19 factors. Both the rotated factor analysis and the scree plot of the eigenvalues indicate that the COVID-19 items load on three factors: items assessing changes in social relationships loaded on a factor labelled ‘COVID-19 social relationships’ (Factor 1); items assessing stress and concerns because of COVID-19 loaded on a factor labelled ‘COVID-19 stress’ (Factor 2); and the single item assessing hope about the end of the pandemic constituted its own factor, labelled ‘COVID-19 hope’ (Factor 3). We report the full procedure used to derive our COVID-19 factor scores in Supplementary Appendix C. Our three COVID-19 factors were rescaled between 0 and 1 to make interpreting coefficients easier across models (COVID-19 social relationships: mean, 0.51, s.d. = 0.16; COVID-19 stress: mean 0.55, s.d. = 0.19; COVID-19 hope: mean 0.45; s.d. = 0.20).

Scores on the CESD-10 range from 0 to 30 (mean 13.07 and s.d. = 6.22), on the STAI-6 from 0 to 18 (mean 8.73 and s.d. = 4.59) and on the PSS-4 from 0 to 16 (mean 7.30 and s.d. = 2.69). Our mental health variables were also rescaled between 0 and 1 for the same purpose.

Table 1 presents the effects of COVID-19 factors on mental health. COVID-19 social relationships (*t*) was positively associated with depression symptoms at time (*t*) (+0.23, *P* < 0.05) and negatively associated with perceived stress at time (*t*) (−0.18, *P* < 0.05). The effects were substantive in size: a negative change in 0.1 point in COVID-19 social relationships measured on a 0 to 1 scale was related to an increase (decrease) of more than one standard deviation in depression (stress) symptoms.

**Table 1:** The effect of coronavirus disease 2019 (COVID-19) factors on mental health^a^

	CESD-10 model, coefficient (s.e.) (*n* = 156)	STAI-6 model, coefficient (s.e.) (*n* = 162)	PSS-4 model, coefficient (s.e.) (*n* = 155)
Mental health (*t* – 1)	0.20*** (0.08)	0.26*** (0.08)	0.26*** (0.09)
COVID-19 social relationships (*t*)	0.23** (0.11)	0.075 (0.13)	−0.18** (0.09)
COVID-19 social relationships social (*t* – 1)	0.12 (0.10)	0.20 (0.13)	−0.17** (0.08)
COVID-19 stress (*t*)	0.27*** (0.08)	0.41*** (0.10)	0.24*** (0.08)
COVID-19 stress (*t* – 1)	−0.03 (0.08)	−0.06 (0.10)	−0.04 (0.07)
COVID-19 hope (*t*)	0.21** (0.09)	0.14 (0.10)	0.14* (0.07)
COVID-19 hope (*t* – 1)	0.01 (0.09)	0.01 (0.10)	−0.00 (0.07)
Gender (Reference: Male)	−0.01 (0.03)	−0.01 (0.04)	0.02 (0.03)
Age	0.00 (0.00)	−0.00 (0.00)	−0.00 (0.00)
Highest education (Reference: Primary school)			
Middle school	−0.00 (0.03)	0.05 (0.04)	−0.02 (0.03)
High school	−0.02 (0.04)	0.01 (0.05)	−0.04 (0.04)
Master/Doctorate	−0.09 (0.07)	−0.06 (0.08)	−0.02 (0.05)
Experienced discrimination (Reference: Yes)	−0.05 (0.04)	−0.04 (0.05)	−0.02 (0.03)
Perceived negative attitude towards Syrian refugees	−0.01 (0.01)	−0.00 (0.01)	0.01*** (0.00)
Unemployed	−0.05 (0.04)	−0.11** (0.05)	0.04 (0.03)
Total household income per month	−0.00 (0.00)	−0.00 (0.00)	−0.00 (0.00)
Years living in Istanbul	0.01* (0.01)	0.01 (0.01)	0.01 (0.01)
Concern about residential status	0.02 (0.02)	0.05** (0.02)	0.03** (0.02)
Constant	−0.13 (0.14)	−0.13 (0.17)	0.19 (0.135)

All values have been rounded to two decimal places. CESD-10, 10-item Center for Epidemiologic Study Depression Scale; STAI-6, 6-item State-Trait Anxiety Inventory; PSS-4, 4-item Perceived Stress Scale.

a. The models are estimated by using the ‘xtreg’ command in Stata with maximum likelihood random-effects estimator. Mental health and COVID-19 factors have been rescaled from 0 to 1.

*** *P* < 0.01, **P < 0.05, *P < 0.10.

Concerns about restrictions, living situation and financial problems because of COVID-19, i.e. COVID-19 stress (*t*), was positively related to all three mental health measures (*P* < 0.01). The effects ranged from +0.24 to +0.41 and were substantive in size. For instance, a negative change of 0.1 point in COVID-19 stress was related to an increase of about one and a half standard deviations in stress and anxiety symptoms.

Feelings of hopelessness about the end of the pandemic, i.e. COVID-19 hope (*t*) was positively associated with both symptoms of depression at time (*t*) (+0.21, *P* < 0.05) and perceived stress at time (*t*) (+0.14, *P* < 0.10). Except for a negative effect of previous changes in the quality of social relationships on stress symptoms, we found no evidence that COVID-19 factors at time (*t* – 1) influence scores on the CESD-10, STAI-6 and PSS-4 at time (*t*).

Estimates for some sociodemographic variables reported meaningful effects on mental health. In particular, higher perception of Turkish society's negative attitude towards Syrian refugees was positively associated with stress symptoms, longer time spent in Istanbul was positively associated with depression symptoms and greater concern about stability of residential status was positively associated with both anxiety and stress symptoms.

Unlike some previous research mentioned above, our data do not show any statistically significant association between gender (or other basic sociodemographics such as age and education) and mental health. However, given gender differences in depression, in supplementary analyses we tested for an interaction effect between COVID-19 factors – at both time (*t*) and (*t* – 1) – and female gender. We fully report these additional analyses in Supplementary Appendix D because in all instances but one the interaction is statistically insignificant. The only exception is given by the interaction with COVID-19 hope (*t* – 1) which is positive and significant at least at *P* < 0.05 in all three models, suggesting that higher feelings of hopelessness about the end of the pandemic in the previous wave are associated with higher symptoms of CESD-10, STAI-6 and PSS-4 in the current wave among women.

Table 2 examines the effects of mental health on COVID-19 factors. Full analyses with sociodemographic variables are presented in Supplementary Appendix E. Depressive symptoms at time (*t*) were positively associated with all COVID-19 factors at time (*t*) and effects ranged from +0.12 on COVID-19 social relationships to +0.25 on COVID-19 stress, namely, between about two-thirds of one standard deviation and one standard deviation in COVID-19 factors. Anxiety symptoms at time (*t*) were positively associated with COVID-19 stress (+0.22, *P* < 0.01), namely, almost one standard deviation in COVID-19 stress. Moreover, stress symptoms at time (*t*) were positively associated with both COVID-19 stress (+0.23, *P* < 0.01) and COVID-19 hope (+0.16, *P* < 0.10), but were negatively associated with COVID-19 social relationships (−0.13, *P* < 0.05). Except for a positive effect of anxiety symptoms at time (*t* – 1) on COVID-19 stress (*t*) (+0.11, *P* < 0.10), there was no evidence that mental health symptoms at time (*t* – 1) influenced COVID-19 factors at time (*t*).

**Table 2:** The effect of mental health on coronavirus disease 2019 (COVID-19) factors^a^

	COVID-19 Social models, coefficient (s.e.)	COVID-19 Stress models, coefficient (s.e.)	COVID-19 Hope models, coefficient (s.e.)
CESD-10 (*t*)	0.12** (0.06)	0.25*** (0.07)	0.18** (0.07)
CESD-10 (*t* – 1)	0.09 (0.05)	−0.06 (0.07)	−0.04 (0.07)
STAI-6 (*t*)	0.03 (0.05)	0.22*** (0.05)	0.08 (0.06)
STAI-6 (*t* – 1)	0.06 (0.05)	0.11* (0.06)	−0.03 (0.07)
PSS-4 (*t*)	−0.13** (0.07)	0.23*** (0.08)	0.16* (0.09)
PSS-4 (*t* – 1)	0.03 (0.08)	0.12 (0.09)	−0.03 (0.10)

CESD-10, 10-item Center for Epidemiologic Study Depression Scale; STAI-6, 6-item State-Trait Anxiety Inventory; PSS-4, 4-item Perceived Stress Scale.

a. The models are estimated by using the ‘xtreg’ command in Stata with maximum likelihood random-effects estimator. Entries come from nine models (three models for each COVID-19 factor, one per mental health factor). *n* = 156 for models with CESD-10; *n* = 162 for models with STAI-6; *n* = 155 for models with PSS-4. Mental health and COVID-19 factors have been rescaled from 0 to 1. The analyses include: gender, age, education, employment status, total monthly household income, years living in Istanbul, experienced discrimination, perception of Turkish society's negative attitude towards Syrian refugees, concern about stability of residential status in Turkey, and previous levels of COVID-19 factors as well as alternative COVID-19 factors’ current and previous levels. Models with sociodemographic factors displayed are reported in Supplementary Appendix E.

***P < 0.01, **P < 0.05, *P < 0.10.

As mentioned earlier, to compare differences in the effects shown above, we use AIC. If the model in which a given mental health measure is the dependent variable has a smaller AIC than the model in which a given COVID-19 factor is the dependent variable, we can conclude that the former model fits the data better than the latter model. Indeed, this is the case for CESD-10 and STAI-6, where AIC is smaller in the analyses of the effects of COVID-19 factors on CSED-10 and STAI-6. For PSS-4, the model in which COVID-19 hope is the dependent variable has the smaller AIC. Thus, we can conclude that COVID-19 factors are better at predicting symptoms of depression and anxiety, whereas symptoms of stress are better at predicting hopelessness about the pandemic. AIC values are presented in Table 3.

**Table 3:** Akaike Information Criterion (AIC) for model comparisons

	AIC
*Depression (CESD-10)*	
COVID-19 factors → CESD-10	−80.6478^a^
CESD-10 → COVID-19 social relationships factor	−181.9183
CESD-10 → COVID-19 stress factor	−94.8846
CESD-10 → COVID-19 hope factor	−107.5108
*Anxiety (STAI-6)*	
COVID-19 factors → STAI-6	−11.8631^b^
STAI-6 → COVID-19 social relationships factor	−168.4335
STAI-6 → COVID-19 stress factor	−113.6477
STAI-6 → COVID-19 hope factor	−89.9590
*Stress (PSS-4)*	
COVID-19 factors → PSS-4	−121.3055
PSS-4 → COVID-19 social relationships factor	−167.9883
PSS-4 → COVID-19 stress factor	−127.8890
PSS-4 → COVID-19 hope factor	−97.2857^c^

COVID-19, coronavirus disease 2019; CESD-10, 10-item Center for Epidemiologic Study Depression Scale; STAI-6, 6-item State-Trait Anxiety Inventory; PSS-4, 4-item Perceived Stress Scale.

a. Lowest AIC value in the depression (CESD-10) models.

b. Lowest AIC value in the anxiety (STAI-6) models.

c. Lowest AIC value in the stress (PSS-4) models.

## Discussion

### Main findings

Although it is now well documented that the COVID-19 pandemic has had an adverse effect on psychiatric symptoms, research examining the impact of the pandemic on the mental health of refugees is still limited. Utilising a two-wave panel study data-set obtained throughout the summer of 2020, we documented an association between COVID-19 stressors and the mental health of Syrian refugees living in Istanbul. The present study is important in demonstrating that, consistent with other recent research examining the effects of the COVID-19 pandemic on mental health,^4,35^ perceptions about COVID-19 policy responses are associated with levels of stress, depression and anxiety in refugees; further, changes in symptoms appear to influence perceptions of the pandemic.

More specifically, we found that changes in the quality of social relationships, pandemic-related stress and feelings of hopelessness about the end of the pandemic were associated with depressive symptoms. In addition, the COVID-19 stress factor was significantly associated with levels of anxiety and perceived stress. Further, we found an association between levels of anxiety in the previous wave and the COVID-19 stress factor in the subsequent wave. This suggests that mental health symptoms, at least for anxiety, have an impact on pandemic-related stress. But this is the only instance in our data where we can conclude that mental illness symptoms affect COVID-19 stressors, as supported in analyses that we conducted using AIC (see above). Finally, contrary to findings in the broader literature, we found only limited evidence of an effect of gender on COVID-19 factors, indicating that in our sample the pandemic-related stresses experienced by both males and females may have overridden possible gender differences.

### Interpretation of our findings

The results of our study highlight important issues that merit further discussion. The first point involves the Syrian refugees’ concerns about restrictions on leaving home, their living situation and financial problems because of COVID-19, and their resultant levels of anxiety. As noted earlier, Syrians’ access to the Turkish labour market has been limited, confining refugees to informal employment. At the same time, the pandemic has inflicted disproportionate health and economic risks on those employed in informal jobs and has exacerbated their vulnerabilities. Many informal jobs provide essential services that involve interaction with other people, where it is more difficult to effectively implement social distancing measures, putting informal workers at high risk of contracting COVID-19. In addition, informal jobs are often excluded from the government's wage subsidy schemes and are not covered by the mitigation policies. Finally, the measures adopted to contain the economic effect of the pandemic place informal workers at higher risk of losing their jobs. For informal workers, job and income insecurity, coupled with a higher risk of contracting COVID-19, are likely to increase the refugees’ vulnerability and adversely affect their mental health.^36^

Financial stress is particularly important because of its links to the related housing problems of refugees in Turkey. Adequate housing is an issue of finding not only a physical shelter, but also a place that ‘protects privacy, contributes to physical and psychological well-being and supports the development and social integration of its inhabitants’.^37,38^ Thus, having adequate housing is considered an important social determinant of mental health by the WHO.^39^ Unable to afford adequate housing, however, many refugees in Turkey live in poor and overcrowded flats.^40^ Financial problems add further stress to the refugees’ housing problems. Insecurity of tenure and the risk of losing their housing increases their vulnerability and adds to their anxiety.

Another point that warrants discussion concerns the negative impact of the government's COVID-19 restriction measures on the quality of the refugees’ social relationships with family, friends and with Turkish society. This is an important issue not only because it affects refugees’ integration into Turkish society, but also because of its links with mental health.^41^ Concepts such as integration, harmonisation, social cohesion and adaptation by refugees have been central to discussions about Syrians in Turkey. In the absence of a universal definition, integration is generally accepted as a dynamic and two-way process that requires participation of both the host society and immigrants/refugees. Researchers often emphasise strengthening of social relations, interactions and ties as important aspects of social cohesion and integration.^42^

COVID-19 measures have taken away opportunities where the two communities met including schools, universities and non-governmental organisations (NGOs). Given the further social distance between the two groups necessitated by these measures, this will have adverse effects on refugees’ participation in the social, cultural and economic life of their community. As earlier studies showed,^43^ lack of social integration can lead to increased stress and poor psychological well-being, aggravating refugees’ mental health problems.

The third point involves the implications of our findings for the trauma and life challenges experienced by Syrian refugees prior to the pandemic. The high levels of psychiatric symptoms among refugees are well documented in the literature. Wars and conflicts are often a life-changing trauma for those who are displaced. Researchers have found declines in mental well-being and, in particular, a rise in depression even in post-traumatic periods.^44^ Indeed, investigators have reported that one-quarter to one-half of refugee adults have clinically significant levels of depression.^45^ Studies focusing on refugees in Turkey also confirm this statistic: Syrian refugees in camps and schools have been found to exhibit high levels of anxiety and depression.^46,47^ In Turkey, most refugees live outside of the camps, and some scholars posit that better mental health of refugees living in cities is the result of them having more job opportunities, alternative accommodation and additional societal support.^47^ Our findings are consistent with this, showing increased anxiety and depression as job opportunities decrease and living standards deteriorate with the pandemic. The present study is also consistent with earlier research linking working and living conditions to mental well-being.

### Limitations

We should note four limitations of this study. First, our panel data-set includes only two waves that were collected within a 2-month interval. Thus, although we can examine short-term effects of the pandemic on mental health, we do not yet understand the longer-term effects. Second, because we received funding for our project after COVID-19 had begun, we can only compare the effects on Syrian refugees’ mental health between two periods within the first COVID-19 wave, but not before the pandemic. Third, our analyses are based on a representative sample of Syrian refugees living outside of camps; thus, we do not know whether our conclusions generalise to refugees housed in camps. Nevertheless, we can speculate that because refugees living outside of camps have easier access to the labour market, the effects of the outcomes of COVID-19 restrictions might be greater for this group. Finally, given space constraints, we could not include in our survey questions concerning worry about illness or death, experience of loss, worry about family members in the home country, or PTSD, all of which might be relevant to our study.

### Implications

Despite these limitations, the present study is important in demonstrating that, consistent with recent research on the effects of COVID-19 on mental health^4^ in unselected samples, the pandemic is having an adverse effect on levels of depression, anxiety and stress in Syrian refugees, and it will be important that they are provided with services to reduce what may be particularly debilitating consequences of COVID-19.

In this regard, our findings have important implications for the support provided for Syrian refugees in Turkey. First, the Turkish government continues to receive huge sums to meet the needs of the Syrian refugees it hosts. The European Union is considering giving 3.5 billion euros for refugee funding to Turkey in addition to the 6 billion euros it has already committed to.^48^ However, there is a need for more support directed towards refugees’ mental health. In addition, although Syrian refugees have access to health services in Turkey, lack of information and accessibility may prevent them from making use of mental health services. Therefore, the national government should work with local actors to improve information sharing and accessibility of mental health services, and develop tailored psychosocial health interventions by increasing the number of adequately trained staff, which might help reduce stigma.^20,49^ This requires the government to prioritise mental health in their refugee-related policies and funding programmes.

Second, local and international civil society actors have been working with and for Syrian refugees living in camps and cities.^50^ Over the years, their activities have facilitated refugees’ access to healthcare, education, the labour market and, more recently, have supported intercommunal dialogue with NGOs conducting projects on refugees’ mental well-being. M'zah and colleagues suggest that the provision of mental health services in non-medical settings may help reduce stigma.^20^ Therefore, NGOs could integrate a mental health component into their ongoing and upcoming projects as an immediate response to alleviate the mental health impact of the pandemic.

NGOs have stepped in with basic needs support for those refugees exposed to job and income losses prompted by the pandemic. This support should continue until the debilitating effects of the pandemic are over. At the same time, however, the uncertain life situation of refugees should be resolved and the process for entering formal employment should be eased to eliminate refugees’ constant insecurity and fear of the future. The uncertainty keeps refugees from having a normal life and will continue to adversely affect their mental health.

## Data Availability

The data that support the findings of this study are available on request from the corresponding author, L.B. The data are not publicly available yet because of restrictions related to publication commitments of the project's research outputs.

## Supplementary material

For supplementary material accompanying this paper visit http://dx.doi.org/10.1192/bjo.2021.1009. click here to view supplementary material

## Appendix

### Life changes because of coronavirus disease 2019 (COVID-19)

**Table:**

During the past 2 weeks …	Response options
… has the quality of the relationships between you and members of your family changed?	1, a lot worse; 2, a little worse; 3, about the same; 4, a little better; 5, a lot better	
… has the quality of your relationships with your friends changed?		
… has the quality of your relationships with the Turkish community changed?		
… how stressful have these changes in social contacts been for you?	1, not at all; 2, slightly; 3, moderately; 4, very; 5, extremely	
… to what degree have changes related to the Coronavirus/COVID-19 crisis in your area created financial problems (such as closure of business, salary cut, loss of job, etc.) for you or your family?		
… how stressful have the restrictions on leaving home been for you?		
… to what degree are you concerned about the stability of your living situation?		
… how hopeful are you that the Coronavirus/COVID-19 crisis in your area will end soon?		
