# Supplementary material for: Effects of COVID-19-related life changes on mental health in Syrian refugees in Turkey
Source: BJPsych Open. 2021 Oct 1;7(6):e182. doi: 10.1192/bjo.2021.1009 (PMC8503067; doi:10.1192/bjo.2021.1009)
Supplement: Supplementary file 1 [file S2056472421010097sup001.docx]

**Online Appendix for “The Effects of COVID-19 Life Changes on Mental Health of Syrian Refugees in Turkey”**

**Appendix A: Survey Questions on Mental Health**

**Appendix B: Descriptive Statistics**

**Appendix C: Factor Analysis of COVID-19 Life Changes**

**Appendix D: Analyses of the Interaction between COVID-19 Factors and Sex on Mental Health**

**Appendix E: Analyses of the Effects of Mental Health on COVID-19 Factors**

**Appendix A: Survey Questions on Mental Health**

**CESD-10**

**Below is a list of ways people sometimes feel or behave. For each item, please think back and indicate how often or how consistently you have felt or behaved this way during THE PAST TWO WEEKS [INTERVIEWER, CIRCLE THE RESPONSE THAT APPLIES].**

_____________________________________________________________________

During the past two weeks:

0 = RARELY OR NONE OF THE TIME (less than 1 day)

1 = SOME OR A LITTLE OF THE TIME (1 - 2 days)

2 = OCCASIONALLY OR A MODERATE AMOUNT OF TIME (3 - 4 days)

3 = MOST OR ALL OF THE TIME (5-7 days)

_____________________________________________________________________

| 1. I was bothered by things that usually don’t bother me. | 0 1 2 3 |
| --- | --- |
| 2. I had trouble keeping my mind on what I was doing. | 0 1 2 3 |
| 3. I felt depressed. | 0 1 2 3 |
| 4. I felt that everything I did was an effort. | 0 1 2 3 |
| 5. I felt hopeful about the future. | 0 1 2 3 |
| 6. I felt fearful. | 0 1 2 3 |
| 7. My sleep was restless. | 0 1 2 3 |
| 8. I was happy. | 0 1 2 3 |
| 9. I felt lonely. | 0 1 2 3 |
| 10. I could not get "going". | 0 1 2 3 |

**STAI-6**

**A number of statements that people use to describe themselves are given below. Read each statement and then fill in the circle with the number which indicates how you have been feeling over the PAST TWO WEEKS. There are no right or wrong answers. Please do not spend too much time on any one statement, but give the answer that seems to *best* describe how you have been feeling. [INTERVIEWER, CIRCLE THE RESPONSE THAT APPLIES].**

During the past two weeks:

0 = NEVER 1 = SOMETIMES 2 = OFTEN 3 = ALMOST ALWAYS

| 1. I have been feeling calm. | 0 1 2 3 |
| --- | --- |
| 2. I have been feeling tense. | 0 1 2 3 |
| 3. I have been feeling relaxed. | 0 1 2 3 |
| 4. I have been feeling upset. | 0 1 2 3 |
| 5. I have been feeling content. | 0 1 2 3 |
| 6. I have been feeling worried. | 0 1 2 3 |

**PSS-4**

**The questions in this scale ask you about your feelings and thoughts during the PAST TWO WEEKS. In each case, you will be asked to indicate how often you felt or thought a certain way.**

For each question, choose from the following alternatives:

0 = never 1 = almost never 2 = sometimes 3 = fairly often 4 = very often

| 1. How often have you felt that you were unable to control the important things in your life? | 0 1 2 3 4 |
| --- | --- |
| 2. How often have you felt confident about your ability to handle your personal problems? | 0 1 2 3 4 |
| 3. How often have you felt that things were going your way? | 0 1 2 3 4 |
| 4. How often have you felt difficulties were piling up so high that you could not overcome them? | 0 1 2 3 4 |

**Appendix B: Descriptive Statistics**

**Table S1. Summary Statistics Main Variables (Wave 1)**

|  | Mean | Std. Dev. | Min. | Max. | N |
| --- | --- | --- | --- | --- | --- |
| CESD-10 | 0.47 | 0.21 | 0 | 1 | 273 |
| STAI-6 | 0.51 | 0.25 | 0 | 1 | 288 |
| PSS-4 | 0.49 | 0.15 | 0 | 1 | 288 |
| Social | 0.49 | 0.16 | 0 | 1 | 278 |
| Stress | 0.56 | 0.20 | 0 | 1 | 278 |
| Hope | 0.43 | 0.21 | 0 | 1 | 278 |

**Table S2. Summary Statistics Main Variables (Wave 2)**

|  | Mean | Std. Dev. | Min. | Max. | N |
| --- | --- | --- | --- | --- | --- |
| CESD-10 | 0.39 | 0.20 | 0 | 1 | 196 |
| STAI-6 | 0.44 | 0.26 | 0 | 1 | 205 |
| PSS-4 | 0.41 | 0.18 | 0 | 1 | 201 |
| Social | 0.54 | 0.16 | 0 | 1 | 193 |
| Stress | 0.53 | 0.19 | 0 | 1 | 193 |
| Hope | 0.48 | 0.20 | 0 | 1 | 193 |

**Table S3. Summary Statistics Control Variables**

|  | Mean | Std. Dev. | Min. | Max. | N |
| --- | --- | --- | --- | --- | --- |
| Sex | 1.5 | 0.50 | 1 | 2 | 512 |
| Age | 31.99 | 10.62 | 18 | 69 | 512 |
| Highest education | 1.99 | 0.91 | 1 | 4 | 512 |
| Experience of discrimination | 1.75 | 0.43 | 1 | 2 | 512 |
| Perceived attitude towards Syrian refugees | 4.49 | 3.28 | 0 | 10 | 501 |
| Unemployed | 0.18 | 0.38 | 0 | 1 | 512 |
| Total household income monthly | 2512 | 1281 | 0 | 8000 | 448 |
| Years living in Istanbul | 5.58 | 1.99 | 1 | 10 | 512 |
| Concern about residential status | 2.28 | 0.95 | 0 | 4 | 501 |

**Appendix C: Factor Analysis of COVID-19 Life Changes**

**COVID-19 questions (in the past two weeks):**

Q1 = how stressful have the restrictions on leaving home been for you?

Q2 = has the quality of the relationships between you and members of your family changed?

Q3 = has the quality of your relationships with your friends changed?

Q4 = has the quality of your relationships with the Turkish community changed?

Q5 = how stressful have these changes in social contacts been for you?

Q6 = to what degree have changes related to the Coronavirus/COVID-19 crisis in your area created financial problems (such as closure of business, salary cut, loss of job, etc.) for you or your family?

Q7 = to what degree are you concerned about the stability of your living situation?

Q8 = how hopeful are you that the Coronavirus/COVID-19 crisis in your area will end soon?

**Step 1. Compute factor analysis with principle-component factor method**

Factor analysis/correlation Number of obs = 471

Method: principal-component factors Retained factors = 3

Rotation: (unrotated) Number of params = 21

--------------------------------------------------------------------------

Factor | Eigenvalue Difference Proportion Cumulative

-------------+------------------------------------------------------------

Factor1 | 2.56247 0.92268 0.3203 0.3203

Factor2 | 1.63979 0.62471 0.2050 0.5253

Factor3 | 1.01508 0.23733 0.1269 0.6522

Factor4 | 0.77775 0.14749 0.0972 0.7494

Factor5 | 0.63026 0.08371 0.0788 0.8282

Factor6 | 0.54655 0.06966 0.0683 0.8965

Factor7 | 0.47689 0.12568 0.0596 0.9561

Factor8 | 0.35121 . 0.0439 1.0000

--------------------------------------------------------------------------

LR test: independent vs. saturated: chi2(28) = 772.98 Prob>chi2 = 0.0000

Factor loadings (pattern matrix) and unique variances

-----------------------------------------------------------

Variable | Factor1 Factor2 Factor3 | Uniqueness

-------------+------------------------------+--------------

q1 | 0.5583 0.4094 0.3702 | 0.3837

q2 | 0.6266 -0.4925 -0.0274 | 0.3641

q3 | 0.7279 -0.4891 -0.0342 | 0.2298

q4 | 0.6915 -0.4248 -0.1978 | 0.3022

q5 | 0.5062 0.5611 0.2618 | 0.3603

q6 | 0.6042 0.3315 -0.1786 | 0.4932

q7 | 0.4782 0.5225 -0.1330 | 0.4806

q8 | 0.0131 -0.3349 0.8479 | 0.1687

-----------------------------------------------------------

**Step 2. Screeplot**

**Step 3. Rotate**

Factor analysis/correlation Number of obs = 471

Method: principal-component factors Retained factors = 3

Rotation: orthogonal varimax (Kaiser off) Number of params = 21

--------------------------------------------------------------------------

Factor | Variance Difference Proportion Cumulative

-------------+------------------------------------------------------------

Factor1 | 2.17293 0.21649 0.2716 0.2716

Factor2 | 1.95644 0.86849 0.2446 0.5162

Factor3 | 1.08796 . 0.1360 0.6522

--------------------------------------------------------------------------

LR test: independent vs. saturated: chi2(28) = 772.98 Prob>chi2 = 0.0000

Rotated factor loadings (pattern matrix) and unique variances

-----------------------------------------------------------

Variable | Factor1 Factor2 Factor3 | Uniqueness

-------------+------------------------------+--------------

q1 | 0.1185 0.7543 0.1825 | 0.3837

q2 | 0.7902 0.0343 0.1017 | 0.3641

q3 | 0.8675 0.0989 0.0886 | 0.2298

q4 | 0.8276 0.0748 -0.0848 | 0.3022

q5 | 0.0036 0.7991 0.0332 | 0.3603

q6 | 0.2941 0.5686 -0.3114 | 0.4932

q7 | 0.0722 0.6394 -0.3245 | 0.4806

q8 | 0.0722 0.0128 0.9088 | 0.1687

-----------------------------------------------------------

Factor rotation matrix

-----------------------------------------

| Factor1 Factor2 Factor3

-------------+---------------------------

Factor1 | 0.7727 0.6323 -0.0559

Factor2 | -0.6120 0.7187 -0.3301

Factor3 | -0.1686 0.2892 0.9423

-----------------------------------------

**Step 4. Sort Rotated Loadings**

Rotated factor loadings (pattern matrix) and unique variances sorted

----------------------------------------------------------

Variable | Factor1 Factor2 Factor3 | Uniqueness

-------------+------------------------------+-------------

q3 | 0.8675 0.0989 0.0886 | 0.2298

q4 | 0.8276 0.0748 -0.0848 | 0.3022

q2 | 0.7902 0.0343 0.1017 | 0.3641

q5 | 0.0036 0.7991 0.0332 | 0.3603

q1 | 0.1185 0.7543 0.1825 | 0.3837

q7 | 0.0722 0.6394 -0.3245 | 0.4806

q6 | 0.2941 0.5686 -0.3114 | 0.4932

q8 | 0.0722 0.0128 0.9088 | 0.1687

----------------------------------------------------------

**Step 5. Predict Factors**

. predict factor1 factor2 factor3

(regression scoring assumed)

Scoring coefficients (method = regression; based on varimax rotated factors)

--------------------------------------------

Variable | Factor1 Factor2 Factor3

-------------+------------------------------

q1 | -0.04590 0.42266 0.24905

q2 | 0.37732 -0.06908 0.06001

q3 | 0.40770 -0.04448 0.05085

q4 | 0.39990 -0.07189 -0.11316

q5 | -0.10025 0.44544 0.11908

q6 | 0.08811 0.24350 -0.24566

q7 | -0.02871 0.30911 -0.23908

q8 | -0.01183 0.09802 0.85423

--------------------------------------------

**Appendix D: Analyses of the Interaction between COVID-19 Factors and Sex on Mental Health**

**Table S4. The Effects of COVID-19 Factors on Mental Health: Sex Differences**

|  | **CESD-10**  **Model** | **STAI-6**  **Model** | **PSS-4**  **Model** |
| --- | --- | --- | --- |
|  |  |  |  |
| Mental Health (t-1) | 0.162^**^ | 0.279^***^ | 0.236^***^ |
|  | (0.0744) | (0.0812) | (0.0878) |
|  |  |  |  |
| COVID-19  Social Relationships (t) | 0.405^***^ | 0.173 | -0.101 |
|  | (0.155) | (0.167) | (0.122) |
|  |  |  |  |
| Female | -0.0995 | -0.275 | 0.159 |
|  | (0.166) | (0.201) | (0.152) |
|  |  |  |  |
| **Female # COVID-19 Social (t)** | **-0.240** | **-0.170** | **-0.154** |
|  | **(0.196)** | **(0.226)** | **(0.162)** |
|  |  |  |  |
| COVID-19  Social Relationships (t-1) | 0.0987 | 0.0573 | -0.131 |
|  | (0.128) | (0.160) | (0.110) |
|  |  |  |  |
| **Female # COVID-19 Social (t-1)** | **0.0531** | **0.290** | **-0.0452** |
|  | **(0.181)** | **(0.216)** | **(0.158)** |
|  |  |  |  |
| COVID-19 Stress (t) | 0.253^**^ | 0.433^***^ | 0.230^**^ |
|  | (0.101) | (0.126) | (0.0955) |
|  |  |  |  |
| **Female # COVID-19 Stress (t)** | **0.0683** | **-0.0936** | **-0.0258** |
|  | **(0.159)** | **(0.195)** | **(0.146)** |
|  |  |  |  |
| COVID-19  Stress (t-1) | -0.0942 | -0.165 | 0.0216 |
|  | (0.116) | (0.137) | (0.102) |
|  |  |  |  |
| **Female # COVID-19 Stress (t-1)** | **0.155** | **0.152** | **-0.129** |
|  | **(0.159)** | **(0.196)** | **(0.144)** |
|  |  |  |  |
| COVID-19 Hope (t) | 0.292^***^ | 0.171 | 0.216^**^ |
|  | (0.103) | (0.120) | (0.0929) |
|  |  |  |  |
| **Female # COVID-19 Hope (t)** | **-0.225** | **-0.0581** | **-0.146** |
|  | **(0.178)** | **(0.211)** | **(0.154)** |
|  |  |  |  |
| COVID-19 Hope (t-1) | -0.219^*^ | -0.261^*^ | -0.144 |
|  | (0.116) | (0.134) | (0.0954) |
|  |  |  |  |
| **Female # COVID-19 Hope (t-1)** | **0.419^***^** | **0.521^***^** | **0.312^**^** |
|  | **(0.161)** | **(0.192)** | **(0.141)** |
|  |  |  |  |
| Age | 0.000286 | -0.000397 | -0.0000829 |
|  | (0.00141) | (0.00172) | (0.00125) |
|  |  |  |  |
| Highest education |  |  |  |
| (Ref: Primary school) |  |  |  |
|  |  |  |  |
| Middle school | 0.00455 | 0.0584 | -0.0109 |
|  | (0.0333) | (0.0408) | (0.0295) |
|  |  |  |  |
| High school | -0.0132 | 0.0200 | -0.0312 |
|  | (0.0427) | (0.0515) | (0.0373) |
|  |  |  |  |
| Master/Doctorate | -0.0992 | -0.0561 | -0.0175 |
|  | (0.0656) | (0.0737) | (0.0522) |
|  |  |  |  |
| Experienced discrimination | -0.0497 | -0.0413 | -0.0295 |
| (Ref: Yes) | (0.0363) | (0.0443) | (0.0316) |
|  |  |  |  |
| Perceived negative attitude towards Syrian refugees | -0.00771 | -0.00627 | 0.0122^***^ |
|  | (0.00487) | (0.00606) | (0.00440) |
|  |  |  |  |
| Unemployed | -0.0583 | -0.118^**^ | 0.0365 |
|  | (0.0387) | (0.0492) | (0.0335) |
|  |  |  |  |
| Total household income per month | -0.0000102 | -0.0000216^*^ | -0.00000606 |
|  | (0.0000111) | (0.0000130) | (0.00000968) |
|  |  |  |  |
| Years living in Istanbul | 0.0165^**^ | 0.0139 | 0.00763 |
|  | (0.00695) | (0.00847) | (0.00629) |
|  |  |  |  |
| Concern about residential status | 0.0176 | 0.0528^**^ | 0.0364^**^ |
|  | (0.0178) | (0.0213) | (0.0154) |
|  |  |  |  |
| Constant | -0.0992 | 0.00651 | 0.106 |
|  | (0.162) | (0.195) | (0.143) |
|  |  |  |  |
| N | 156 | 162 | 155 |

Standard errors in parentheses

^*^ *p* < 0.10, ^**^ *p* < 0.05, ^***^ *p* < 0.01

**Appendix E: Analyses of the Effects of Mental Health on COVID-19 Factors**

**Table S5. The Effects of Depression Symptoms on COVID-19 Factors**

|  | **COVID-19 Social**  **Model** | **COVID-19 Stress**  **Model** | **COVID-19 Hope**  **Model** |
| --- | --- | --- | --- |
|  |  |  |  |
| Social (t-1) | 0.125^*^ | 0.0622 | 0.0507 |
|  | (0.0730) | (0.0973) | (0.0935) |
|  |  |  |  |
| Social (t) |  | -0.117 | 0.106 |
|  |  | (0.105) | (0.101) |
|  |  |  |  |
| Stress (t) | -0.0669 |  | 0.0883 |
|  | (0.0603) |  | (0.0766) |
|  |  |  |  |
| Stress (t-1) | -0.00642 | 0.248^***^ | -0.0386 |
|  | (0.0607) | (0.0777) | (0.0770) |
|  |  |  |  |
| Hope (t) | 0.0657 | 0.0957 |  |
|  | (0.0629) | (0.0830) |  |
|  |  |  |  |
| Hope (t-1) | -0.0928 | -0.164^**^ | 0.453^***^ |
|  | (0.0608) | (0.0799) | (0.0687) |
|  |  |  |  |
| CESD-10 (t) | 0.118^**^ | 0.248^***^ | 0.177^**^ |
|  | (0.0571) | (0.0739) | (0.0721) |
|  |  |  |  |
| CESD-10 (t-1) | 0.0871 | -0.0624 | -0.0403 |
|  | (0.0547) | (0.0728) | (0.0700) |
|  |  |  |  |
| Female (Ref: Male) | 0.0324 | 0.00859 | -0.0131 |
|  | (0.0207) | (0.0276) | (0.0265) |
|  |  |  |  |
| Age | 0.00171^*^ | -0.0000451 | -0.000818 |
|  | (0.00101) | (0.00134) | (0.00129) |
|  |  |  |  |
| Highest education |  |  |  |
| (Ref: Primary school) |  |  |  |
|  |  |  |  |
| Middle school | 0.0727^***^ | 0.0617^*^ | -0.0544^*^ |
|  | (0.0240) | (0.0323) | (0.0311) |
|  |  |  |  |
| High school | 0.0511 | 0.00615 | 0.0382 |
|  | (0.0314) | (0.0418) | (0.0400) |
|  |  |  |  |
| Master/Doctorate | 0.119^**^ | 0.0404 | -0.0470 |
|  | (0.0479) | (0.0645) | (0.0619) |
|  |  |  |  |
| Experienced discrimination |  |  |  |
| (Ref: Yes) | 0.0238 | 0.00180 | 0.0341 |
|  | (0.0271) | (0.0359) | (0.0343) |
|  |  |  |  |
| Perceived negative attitude towards Syrian refugees | -0.00696^**^ | -0.00376 | 0.00908^**^ |
|  | (0.00350) | (0.00467) | (0.00444) |
|  |  |  |  |
| Unemployed | 0.0204 | 0.0650^*^ | 0.0258 |
|  | (0.0277) | (0.0363) | (0.0352) |
|  |  |  |  |
| Total household income per month | -0.0000202^**^ | -0.0000225^**^ | 0.0000102 |
|  | (0.00000788) | (0.0000105) | (0.0000102) |
|  |  |  |  |
| Years living in Istanbul | -0.00275 | -0.000314 | 0.00256 |
|  | (0.00510) | (0.00674) | (0.00647) |
|  |  |  |  |
| Concern about residential status | -0.0419^***^ | 0.0307^*^ | -0.0362^**^ |
|  | (0.0127) | (0.0172) | (0.0164) |
|  |  |  |  |
| Constant | 0.480^***^ | 0.325^**^ | 0.153 |
|  | (0.0906) | (0.128) | (0.124) |
|  |  |  |  |
| N | 156 | 156 | 156 |

Standard errors in parentheses

^*^ *p* < 0.10, ^**^ *p* < 0.05, ^***^ *p* < 0.01

**Table S6. The Effects of Anxiety Symptoms on COVID-19 Factors**

|  | **COVID-19 Social**  **Model** | **COVID-19 Stress**  **Model** | **COVID-19 Hope**  **Model** |
| --- | --- | --- | --- |
|  |  |  |  |
| Social (t-1) | 0.0989 | -0.0910 | -0.0357 |
|  | (0.0779) | (0.0925) | (0.0997) |
|  |  |  |  |
| Social (t) |  | -0.0603 | 0.167^*^ |
|  |  | (0.0929) | (0.0992) |
|  |  |  |  |
| Stress (t) | -0.0430 |  | 0.136 |
|  | (0.0663) |  | (0.0838) |
|  |  |  |  |
| Stress (t-1) | 0.0314 | 0.197^***^ | -0.0661 |
|  | (0.0617) | (0.0715) | (0.0786) |
|  |  |  |  |
| Hope (t) | 0.103^*^ | 0.117 |  |
|  | (0.0611) | (0.0724) |  |
|  |  |  |  |
| Hope (t-1) | -0.135^**^ | -0.195^***^ | 0.466^***^ |
|  | (0.0627) | (0.0737) | (0.0722) |
|  |  |  |  |
| STAI-6 (t) | 0.0287 | 0.219^***^ | 0.0836 |
|  | (0.0484) | (0.0548) | (0.0614) |
|  |  |  |  |
| STAI-6 (t-1) | 0.0644 | 0.107^*^ | -0.0271 |
|  | (0.0518) | (0.0610) | (0.0662) |
|  |  |  |  |
| Female (Ref: Male) | 0.0251 | 0.00409 | -0.0132 |
|  | (0.0217) | (0.0257) | (0.0277) |
|  |  |  |  |
| Age | 0.00208^**^ | 0.000499 | -0.000262 |
|  | (0.00105) | (0.00126) | (0.00135) |
|  |  |  |  |
| Highest education |  |  |  |
| (Ref: Primary school) |  |  |  |
|  |  |  |  |
| Middle school | 0.0552^**^ | 0.0490 | -0.0699^**^ |
|  | (0.0256) | (0.0305) | (0.0326) |
|  |  |  |  |
| High school | 0.0371 | -0.00204 | 0.0131 |
|  | (0.0322) | (0.0383) | (0.0412) |
|  |  |  |  |
| Master/Doctorate | 0.106^**^ | 0.0563 | -0.0647 |
|  | (0.0460) | (0.0552) | (0.0593) |
|  |  |  |  |
| Experienced discrimination |  |  |  |
| (Ref: Yes) | 0.00797 | 0.0107 | 0.0371 |
|  | (0.0283) | (0.0335) | (0.0360) |
|  |  |  |  |
| Perceived negative attitude towards Syrian refugees | -0.00989^***^ | -0.00459 | 0.00877^*^ |
|  | (0.00363) | (0.00438) | (0.00468) |
|  |  |  |  |
| Unemployed | 0.0165 | 0.0677^*^ | -0.0425 |
|  | (0.0307) | (0.0360) | (0.0390) |
|  |  |  |  |
| Total household income per month | -0.0000173^**^ | -0.0000160^*^ | 0.0000146 |
|  | (0.00000811) | (0.00000966) | (0.0000104) |
|  |  |  |  |
| Years living in Istanbul | -0.00375 | -0.00150 | -0.00282 |
|  | (0.00530) | (0.00628) | (0.00675) |
|  |  |  |  |
| Concern about residential status | -0.0441^***^ | 0.0205 | -0.00559 |
|  | (0.0132) | (0.0161) | (0.0174) |
|  |  |  |  |
| Constant | 0.535^***^ | 0.320^***^ | 0.136 |
|  | (0.0950) | (0.120) | (0.132) |
|  |  |  |  |
| N | 162 | 162 | 162 |

Standard errors in parentheses

^*^ *p* < 0.10, ^**^ *p* < 0.05, ^***^ *p* < 0.01

**Table S7. The Effects of Stress Symptoms on COVID-19 Factors**

|  | **COVID-19 Social**  **Model** | **COVID-19 Stress**  **Model** | **COVID-19 Hope**  **Model** |
| --- | --- | --- | --- |
|  |  |  |  |
| Social (t-1) | 0.0988 | 0.0269 | 0.0202 |
|  | (0.0728) | (0.0834) | (0.0920) |
|  |  |  |  |
| Social (t) |  | -0.0546 | 0.212^**^ |
|  |  | (0.0913) | (0.0994) |
|  |  |  |  |
| Stress (t) | -0.0421 |  | 0.200^**^ |
|  | (0.0705) |  | (0.0872) |
|  |  |  |  |
| Stress (t-1) | 0.122^**^ | 0.248^***^ | -0.116 |
|  | (0.0610) | (0.0674) | (0.0771) |
|  |  |  |  |
| Hope (t) | 0.134^**^ | 0.165^**^ |  |
|  | (0.0630) | (0.0716) |  |
|  |  |  |  |
| Hope (t-1) | -0.0755 | -0.0874 | 0.455^***^ |
|  | (0.0627) | (0.0713) | (0.0701) |
|  |  |  |  |
| PSS-4 (t) | -0.134^**^ | 0.227^***^ | 0.157^*^ |
|  | (0.0683) | (0.0765) | (0.0859) |
|  |  |  |  |
| PSS-4 (t-1) | 0.0306 | 0.115 | -0.0268 |
|  | (0.0778) | (0.0881) | (0.0978) |
|  |  |  |  |
| Female (Ref: Male) | 0.0491^**^ | 0.0399 | -0.00663 |
|  | (0.0225) | (0.0258) | (0.0287) |
|  |  |  |  |
| Age | 0.00245^**^ | 0.00105 | -0.00126 |
|  | (0.00104) | (0.00120) | (0.00133) |
|  |  |  |  |
| Highest education |  |  |  |
| (Ref: Primary school) |  |  |  |
|  |  |  |  |
| Middle school | 0.0452^*^ | 0.0547^*^ | -0.0491 |
|  | (0.0255) | (0.0290) | (0.0321) |
|  |  |  |  |
| High school | 0.0252 | 0.00177 | 0.0268 |
|  | (0.0323) | (0.0368) | (0.0406) |
|  |  |  |  |
| Master/Doctorate | 0.0779^*^ | 0.0240 | -0.0347 |
|  | (0.0451) | (0.0518) | (0.0572) |
|  |  |  |  |
| Experienced discrimination |  |  |  |
| (Ref: Yes) | -0.00314 | -0.0340 | 0.0506 |
|  | (0.0277) | (0.0314) | (0.0346) |
|  |  |  |  |
| Perceived negative attitude towards Syrian refugees | -0.00881^**^ | -0.00688 | 0.00632 |
|  | (0.00381) | (0.00437) | (0.00484) |
|  |  |  |  |
| Unemployed | 0.0394 | 0.0691^**^ | -0.0168 |
|  | (0.0290) | (0.0328) | (0.0367) |
|  |  |  |  |
| Total household income per month | -0.0000200^**^ | -0.0000285^***^ | 0.0000177^*^ |
|  | (0.00000821) | (0.00000925) | (0.0000104) |
|  |  |  |  |
| Years living in Istanbul | -0.0107^**^ | -0.00632 | 0.00203 |
|  | (0.00537) | (0.00617) | (0.00683) |
|  |  |  |  |
| Concern about residential status | -0.0215 | 0.0417^***^ | -0.0302^*^ |
|  | (0.0134) | (0.0150) | (0.0168) |
|  |  |  |  |
| Constant | 0.520^***^ | 0.202^*^ | 0.0750 |
|  | (0.0999) | (0.122) | (0.136) |
|  |  |  |  |
| N | 155 | 155 | 155 |

Standard errors in parentheses

^*^ *p* < 0.10, ^**^ *p* < 0.05, ^***^ *p* < 0.01
